# Searching for a second excitation in the inelastic neutron scattering spectrum of a liquid metal: a Bayesian analysis

**DOI:** 10.1038/s41598-021-93452-w

**Published:** 2021-07-07

**Authors:** Alessio De Francesco, Ubaldo Bafile, Alessandro Cunsolo, Luisa Scaccia, Eleonora Guarini

**Affiliations:** 1 CNR-IOM & INSIDE@ILL c/o Operative Group in Grenoble (OGG), F-38042 Grenoble, France; 2 Institut Laue-Langevin (ILL), F-38042 Grenoble, France; 3 Consiglio Nazionale delle Ricerche, Istituto di Fisica Applicata “Nello Carrara”, Via Madonna del Piano 10, I-50019 Sesto Fiorentino, Italy; 4 Department of Physics, University of Wisconsin at Madison, 1150 University Avenue, Madison, WI USA; 5 Dipartimento di Economia e Diritto, Università di Macerata, Via Crescimbeni 20, I-62100 Macerata, Italy; 6 Dipartimento di Fisica e Astronomia, Università di Firenze, Via G. Sansone 1, I-50019 Sesto Fiorentino, Italy

**Keywords:** Condensed-matter physics, Statistical physics, thermodynamics and nonlinear dynamics

## Abstract

When probed at nanometer and picosecond scales, the properties of a liquid present striking analogies with the ones of the corresponding solid, one of the most surprising is the ability of supporting shear wave propagation, as a rigid medium. Although this evidence is being reported by a growing number of terahertz scattering measurements, it remains an open question whether it is universal or rather typical of some liquids only. Furthermore, given its elusive signatures in the scattering signal, the detection of this effect appears as a typical case where an unintentional “bias of confirmation” can mislead experimentalists. We thus decided to use a Bayesian inference approach to achieve a probabilistically grounded and evidence-based lineshape modeling of the inelastic neutron scattering spectra from liquid silver, whose simulated density autocorrelations bear evidence of a shear mode propagation over very short distances. The result of our analysis indicates that the observation of any additional, non-longitudinal, acoustic modes in this simple system goes beyond the accuracy of the used scattering method.

## Introduction

In the last decades, growing experimental evidence is being gathered in support of the similarities between the response of liquid and solid systems over short distances and timescales. Advances in the accuracy of available probes have corroborated the overall perception of such similarity while unraveling more subtle aspects of it.

In general, the distinction between a solid-like (rigid and elastic) and a liquid-like (viscous and dissipative) response of non-gaseous systems becomes increasingly elusive upon approaching microscopic scales.

In fact, in liquids, atoms are so tightly packed that the only movements they can experience are primarily rapid rattling oscillations within their first neighbors cage, which have a direct analogy in the lattice vibrations of an ordered solid. However, it remains an open question to what extent the collective atomic dynamics mirrors this similarity.

For instance, a genuine solid-like aspect of the terahertz acoustic dynamics of some liquids is the onset of a shear mode. However, its appearance in the system response, that is in the spectrum of density fluctuations *S*(*Q*, *E*), where *Q* and *E* can be identified with the amplitude wavevector and energy exchanged in a scattering process^1–7^, is far from obvious. Despite having been reported in a wealth of diverse materials, the experimental observation of this “high-frequency rigidity” of liquids poses severe challenges. To begin with, spectroscopic methods best suited to probe it, such as Inelastic Neutron (INS) and X-ray Scattering (IXS), are intrinsically unfit to assess the transverse polarization of an acoustic mode in *S*(*Q*, *E*), unless sided by a parallel computational effort^8^ able to determine other, more appropriate, correlation functions inaccessible to experiments. Even so, convincing experimental evidence for this effect is often hampered by its loosely resolved and feeble spectral signatures.

In these respects, dealing with molten metals (MMs)^9,10^ offers undoubted advantages. The monatomic character of MMs makes them simple benchmarks of possible universal properties of liquid aggregates, also making computer simulations of their microscopic interactions more straightforward. Furthermore the spectrum of density fluctuations of MMs, as opposed to that of other disordered systems, is featured by well-resolved and intense inelastic peaks and often non-preponderant elastic peak^11^. These are ideal prerequisites to detect weak inelastic features gathering in the low-frequency portion of the spectrum, as is likely the case of transverse acoustic modes in liquids. Yet, literature works reporting the onset of shear modes in the spectrum of MMs^2–4,12,13^ often entailed some stretch of the model’s predictive capabilities, or, perhaps an excessive reliance on the resolution, spectral contrast, or statistical accuracy of the measurement.

The situation appears even more worrisome when considering that the use of lineshape models containing a disproportionate number of free parameters is becoming widespread in this kind of studies.

A natural remedy against this slippery slope would be a more strict adherence to the Occam razor principle or “lex parsimoniae”, which states that among two models providing an equally satisfactory account of some evidence, the simpler one, i.e. the one containing fewer parameters, is always preferable. Bayesian inference methods^14,15^ naturally incorporate this principle while providing criteria to rate the plausibility of a given model based on the experimental outcome. As demonstrated by previous works of our group^16–20^, Bayesian methods can be successfully implemented to achieve a minimally biased and probabilistically grounded modeling of the scattering signal. In this work, the inferential approach rests on the use of a Markov Chain Monte Carlo (MCMC) algorithm endowed with a Reversible Jump (RJ) option^21^. The spectra from the sample are modeled by the sum of *k* Damped Harmonic Oscillator (DHO) profiles and a Lorentzian term, accounting for the inelastic and quasielastic portions of the spectra, respectively. In this description *k* itself is treated as a free model parameter whose optimal value and related posterior distribution are to be determined by the algorithm conditionally on the experimental outcome. Details on this Bayesian inference algorithm are illustrated in detail in Refs.^16,18,22^.

In one of our previous works^16^, the Bayesian inferential method was applied to the modeling of INS spectra of liquid gold and led to an unambiguous and probabilistically well-grounded conclusion: a single longitudinal acoustic mode dominates the inelastic wings of the spectrum of gold. However, the posterior probability of the $k=2$ hypothesis sizably increased with *Q*-increase, becoming non-negligible for $Q\ge$10 $\hbox {nm}^{-1}$. Back at those times, we dismissed the hypothesis of a second inelastic mode for a few reasons. First, upon *Q*-increase, the spectral modes got increasingly damped, also migrating toward the border of the accessible kinematic range. Second, the limited count statistics of measured spectra raised the legitimate suspicion that the alleged emergence of a second inelastic mode at the highest *Q*’s could have been an artifact of the low accuracy in the spectral shape definition.

More recently, some of us measured the INS spectrum of liquid silver and modeled its shape with a Generalized Hydrodynamics (GH) profile^23^. Data allowed only a GH modeling, while other more complex models, like the viscoelastic one, gave non-significant values for additional parameters with respect to the GH lineshape. Once again, no spectral signatures were found of low-frequency spectral features to be possibly assigned to a shear wave propagation. However, the close agreement between experiment and ab initio molecular dynamics (AIMD) computations in an overlapping *Q*-range authorized an analysis of simulations in a larger *Q* range. The outcome of this analysis disclosed new interesting scenarios. Indeed, the modeling of the Fourier transform of *S*(*Q*, *E*) with an exponential series expansion^24–26^, already successfully tested for classical^27–29^ and quantum^30,31^ fluids, strongly indicated that a transverse dispersion branch emerges in liquid silver for $Q\ge$15 $\hbox {nm}^{-1}$. The assignment of a transverse nature to such a mode polarization was granted because of the agreement of the found trend for the excitation frequencies with the peak positions in the transverse current correlation function.

To further elaborate on this interesting result, here we use the mentioned Bayesian methods to rate probabilistically the plausibility of a second, transverse, mode in the INS spectrum of molten silver, previously measured by INS or simulated by ab initio methods^32^.

While pursuing this well-targeted objective, we could benefit from three complementary assets: the direct probe provided by the measurement, the one inherently free from experimental uncertainties bestowed by the simulation, and their unbiased modeling allowed by the Bayesian inferential approach.

## Results and discussion

In the following, we succinctly describe the various steps of our analysis as:

### The model function

The experimental data are given by:

1 $$\begin{aligned} y(Q,E)={\tilde{S}}(Q,E)+\varepsilon (Q,E) \end{aligned}$$

where *y*(*Q*, *E*) are the measured scattering data, $\varepsilon (Q,E)$ are the experimental errors and $E=\hbar \omega$ is the energy transferred from the impinging neutron beam to the sample atoms; ${\tilde{S}}(Q,E)$ is our model function convoluted with the instrument resolution which in the present case is well described by a zero-centred Gaussian function with $\sigma =$1.36 meV:

2 $$\begin{aligned} {\tilde{S}}(Q,E)= \left[ \frac{1}{\sqrt{2\pi }\sigma }\exp {\left( -\frac{E^2}{2\sigma ^2} \right) }\right] \otimes S(Q,E). \end{aligned}$$

The chosen model function is given by:

3 $$\begin{aligned} S(Q,E)=[n(E)+1]{\beta E}\Bigg \{\frac{1}{\pi }\frac{A_0(Q)z_0(Q)}{E^2+z_0^2(Q)} +\sum _{j=1}^k\frac{2}{\pi }\frac{A_j(Q)\Omega ^2_j(Q)\Gamma _j(Q)}{[E^2-\Omega _j^2(Q)]^2+4[\Gamma _j(Q)E]^2}\Bigg \} \end{aligned}$$

where $n(E)=(e^{E/k_{B}T}-1)^{-1}$ is the Bose factor expressing the detailed balance condition (with *T* = 1273 K being the temperature of the sample), while the term enclosed by the curly brackets is the sum of a central Lorentzian term, characterized by parameters $A_0$ and $z_0$, plus *k* DHO doublets. The generic *j*-th DHO is featured by its undamped oscillation frequency $\Omega _j (Q)$, a damping $\Gamma _j (Q)$, and intensity factor $A_j (Q)$.

Although it has been shown^33^ how the representation of Brillouin neutron scattering data in terms of DHO doublets is an oversimplified description lacking accuracy and scientific rigor, it is largely employed to model the dynamic structure factor in liquids and, particularly with noisy data, such description can capture the relevant features of a typical spectrum, especially when the energy transfer range explored is limited. Furthermore, for consistency, we deliberately adopted the same model already used for liquid gold.

The best-fitting of the model to the experimental spectra of liquid silver was performed in the 4–16 $\hbox {nm}^{-1}$ interval with the mentioned MCMC-RJ algorithm above mentioned and which is extensively described elsewhere^16,18,22^.

### The Bayesian inference

The method essentially relies on the Bayes theorem^34^ and so do all inference results derived from the joint posterior distribution of all model parameters. In this work, we are especially interested in the posterior distribution of the parameter *k* which is obtained through a marginalization process of the joint posterior distribution $P(\Theta ,k\vert y)$ where $\Theta =\Theta (\theta _1,\theta _2\dots .\theta _n)$ is a vector whose components are all the lineshape model parameters. In particular, the algorithm identifies the model having the highest posterior probability and also its actual probability. The latter is given by:

4 $$\begin{aligned} P(k|y)=\int _{\Theta }P(\Theta ,k|y)d\Theta \propto \int _{\Theta }P(y|\Theta ,k)P(\Theta ,k)d\Theta =P(k) \int _{\Theta }P(y|\Theta ,k)P(\Theta \vert k)d\Theta = P(y|k)P(k), \end{aligned}$$

where the last equation is obtained using the product rule:

5 $$\begin{aligned} P(y,\Theta \vert k)=P(y\vert \Theta ,k)P(\Theta \vert k) \end{aligned}$$

and introducing the final marginalization integral:

6 $$\begin{aligned} P(y\vert k)=\int _{\Theta }P(y,\Theta \vert k)d\Theta . \end{aligned}$$

Upon assuming, as we did, a uniform prior distribution for each possible model option, i.e. imposing ($P(k=1)=P(k=2)=\dots .P(k=n)$), the posterior probability $P(k\vert y)$ becomes simply proportional to the so-called marginal likelihood. This assumption implies that one refrains from assigning a higher a priori probability to the presence of a second (transverse) mode, despite the more assertive conclusions suggested by the analysis of AIMD results at large *Q*’s. This deliberate choice was adopted to let the inferential process be driven uniquely by the experimental evidence without any external bias.

### Measurement outcome and data analysis results

**Figure 1:**
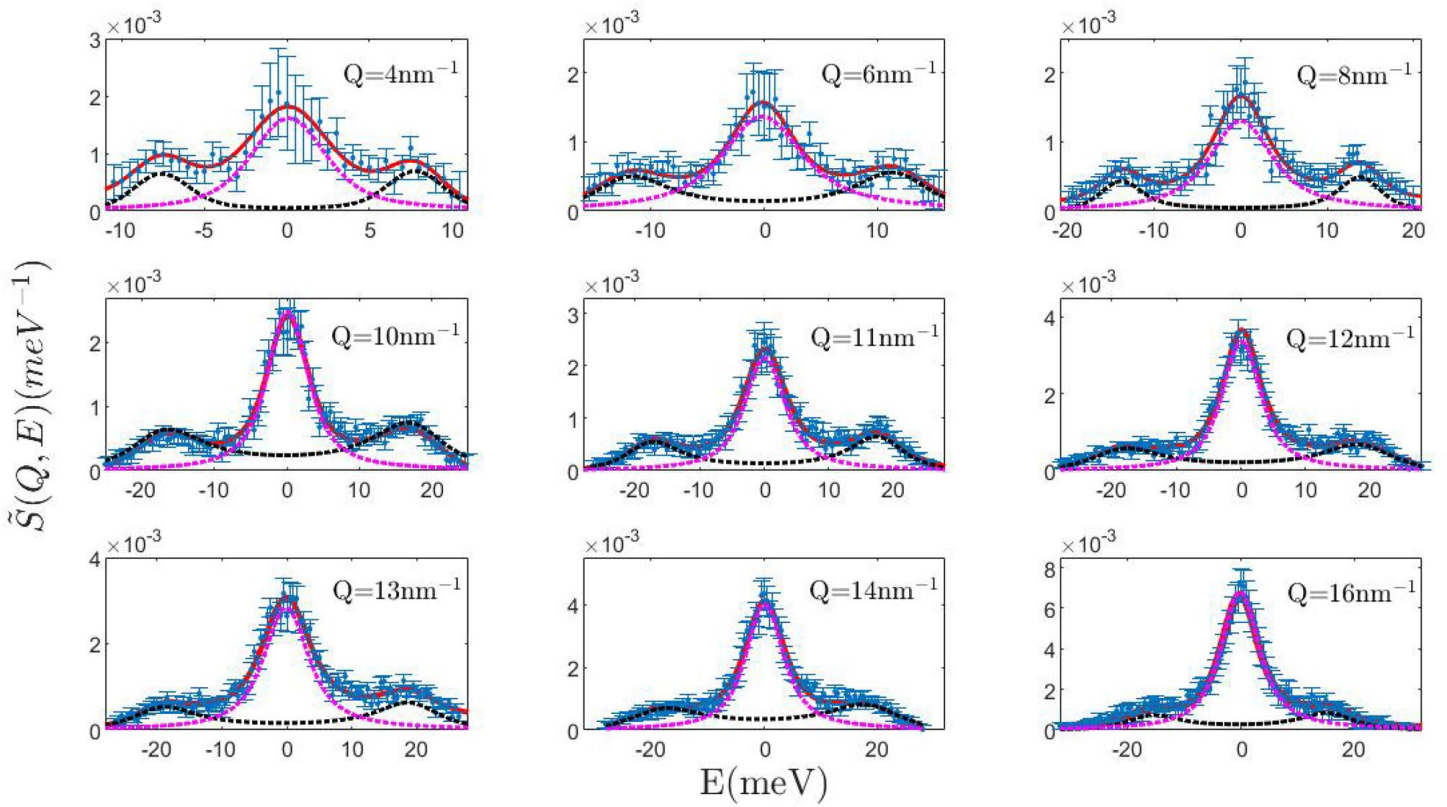
Dynamic structure factor of silver at nine selected *Q* values. The experimental data points are affected by resolution broadening. The corresponding fit obtained by the MCMC-RJ algorithm (solid red line) has been carried out considering the detailed balance asymmetry and finite resolution. The pink and the black dotted lines are the Lorentzian and DHO component respectively.

Figure 1 compares the INS signal collected from the liquid silver sample with the corresponding best-fitting lineshapes privileged by the MCMC-RJ algorithm. It can be readily noticed that, as in our previous INS measurement on a molten metal (gold), the most plausible model features a single inelastic component all over the explored *Q* range. It also appears that this peak’s centroid systematically shifts to higher energy transfers upon *Q* increase up to a maximum and then decreases; the dispersive behavior of this mode, to be discussed further below, urges us to assign this peak to the longitudinal acoustic mode of silver. This assignment also follows from two simple considerations: longitudinal modes, i.e. movements parallel to the exchanged momentum ${\varvec{Q}}$ are those directly coupled with the variable probed by the measurement, i.e. the *S*(*Q*, *E*), and the slope of the low *Q* dispersion leads to estimate a propagation speed close to the speed of sound in silver, i.e. about 2790 m/s.

Overall, data in Fig. 1 highlight, once corrected for the incoherent contribution (see details in Ref.^23^), two distinctive aspects of the spectrum of MMs as compared to the one of other liquid systems, i.e. the persistence of a well-defined triplet shape up to *Q* values approaching the position of the first sharp diffraction maximum, and an inelastic-to-elastic intensity ratio quite larger than in non-metallic liquids. As mentioned in the introductory section, both features seem advantageous when attempting to spot loosely resolved low frequency spectral features, which is how the alleged transverse mode in the spectrum supposedly looks like.

**Figure 2:**
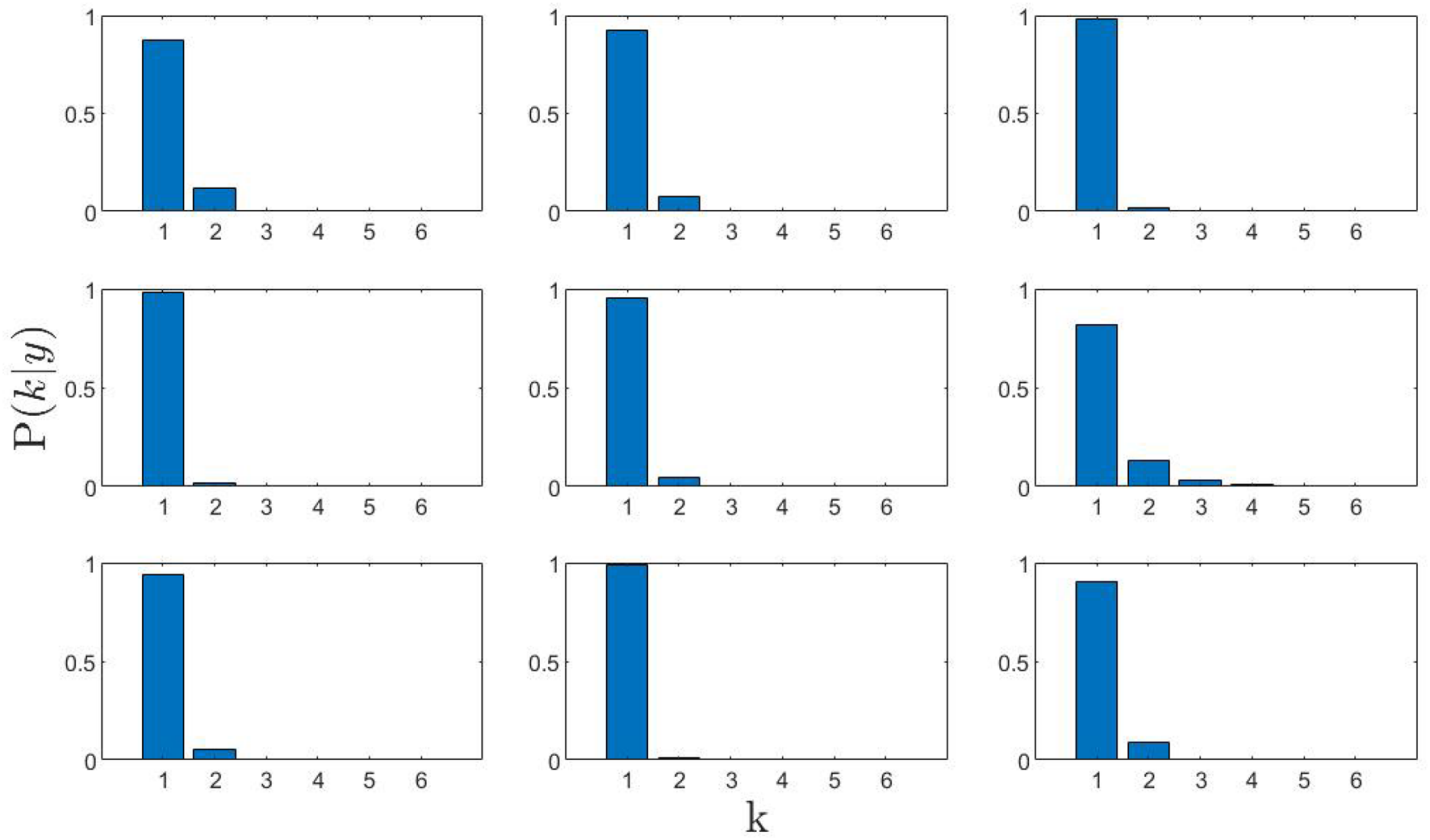
Posterior distribution functions of the number *k* of inelastic components present in the dynamic structure factor spectra shown in Fig. 1. No evidence for a second mode in the spectra is detected as the momentum transfer increases.

As clearly shown by Fig. 2, the posterior probability corresponding to a double excitation hypothesis, $P(k=2\vert y)$, is always negligible, and, at variance with what previously found on liquid gold, it doesn’t seem to depend on *Q* in any systematic fashion, not even at the highest *Q*’s. This partial scrutiny of the measurement outcome urges us to conclude that, albeit AIMD calculated spectral shape bore unquestionable evidence for a double excitation structure, the plausibility of a transverse mode in the measured spectra appears overly slim.

**Figure 3:**
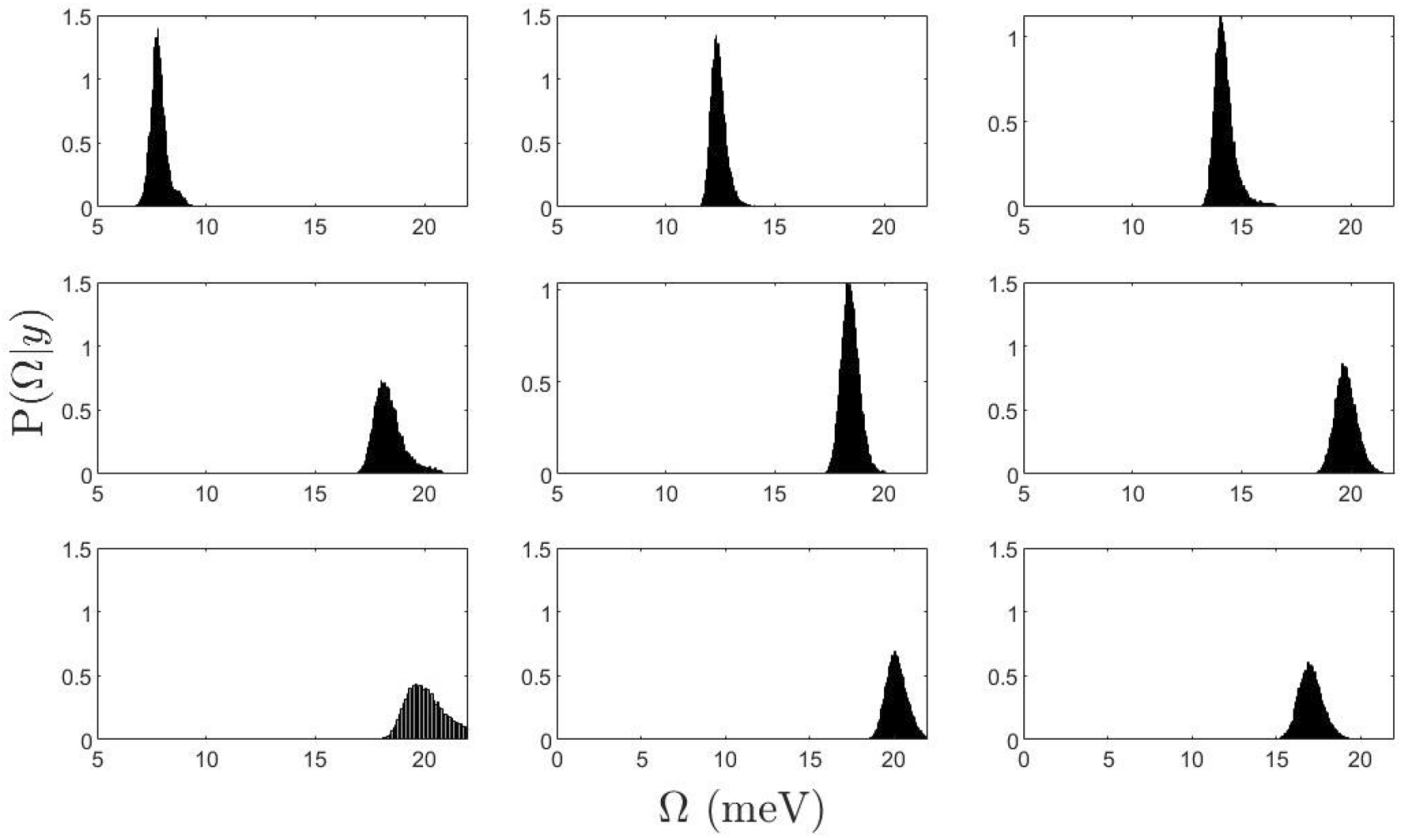
Posterior distribution functions for the undamped excitation frequency $\Omega$ derived from the best fit of the spectra at the same selected *Q* values of Fig. 1.

Figure 3 displays the posterior density distribution drawn by the algorithm for $\Omega (Q)$. The normalized distribution function appears well shaped and unimodal thus confirming the robustness of the single inelastic (longitudinal) mode scenario. Similar figures representing the posterior distributions of other model parameters ($\Gamma (Q),A(Q),z_0(Q),A_0(Q)$) are available in the Supplementary Information (SI).

**Figure 4:**
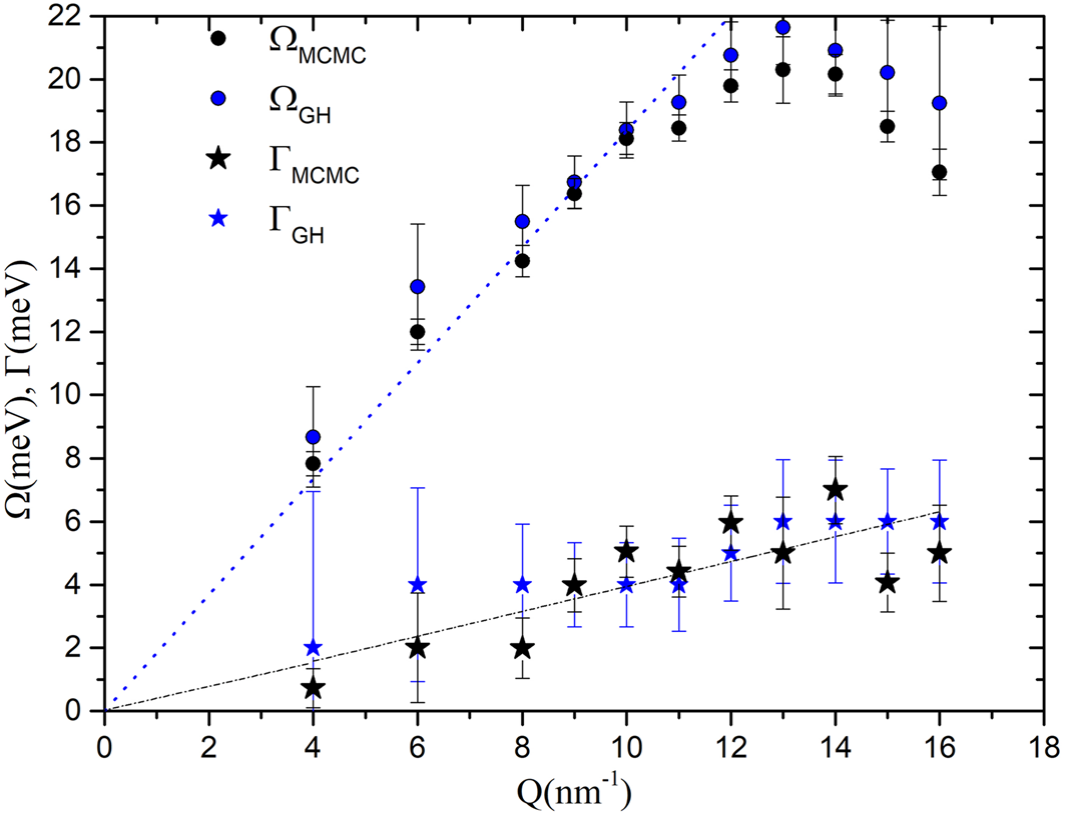
*Q* dependence of the undamped frequency $\Omega$ (circles) and the damping $\Gamma$ (stars) of the longitudinal mode obtained from the fits to the dynamics structure factor by means of the GH model (blue simbols) and the MCMC algorithm (black symbols). The hydrodynamic behaviour for liquid silver (sound speed $\sim$ 2790 m/s) is also shown (blue dotted line). The linear black dotted line is only a guide for the eye.

Figure 4 compares the *Q*-dependencies of the undamped frequency $\Omega (Q)$ and the damping $\Gamma (Q)$ of the longitudinal acoustic mode as obtained either in this work or in Ref.^23^ through a GH modeling of present spectra. The overall agreement between the two curves is more than satisfactory, although absolute values of the excitation frequencies from Ref.^23^ are slightly larger than those estimated by the MCMC algorithm in the present work, such small difference being very likely due to the current use of the DHO model. A close agreement is also obtained for the halfwidth of the central Lorentzian component (see Supplementary Fig. S5).

### The transverse mode hypothesis

**Figure 5:**
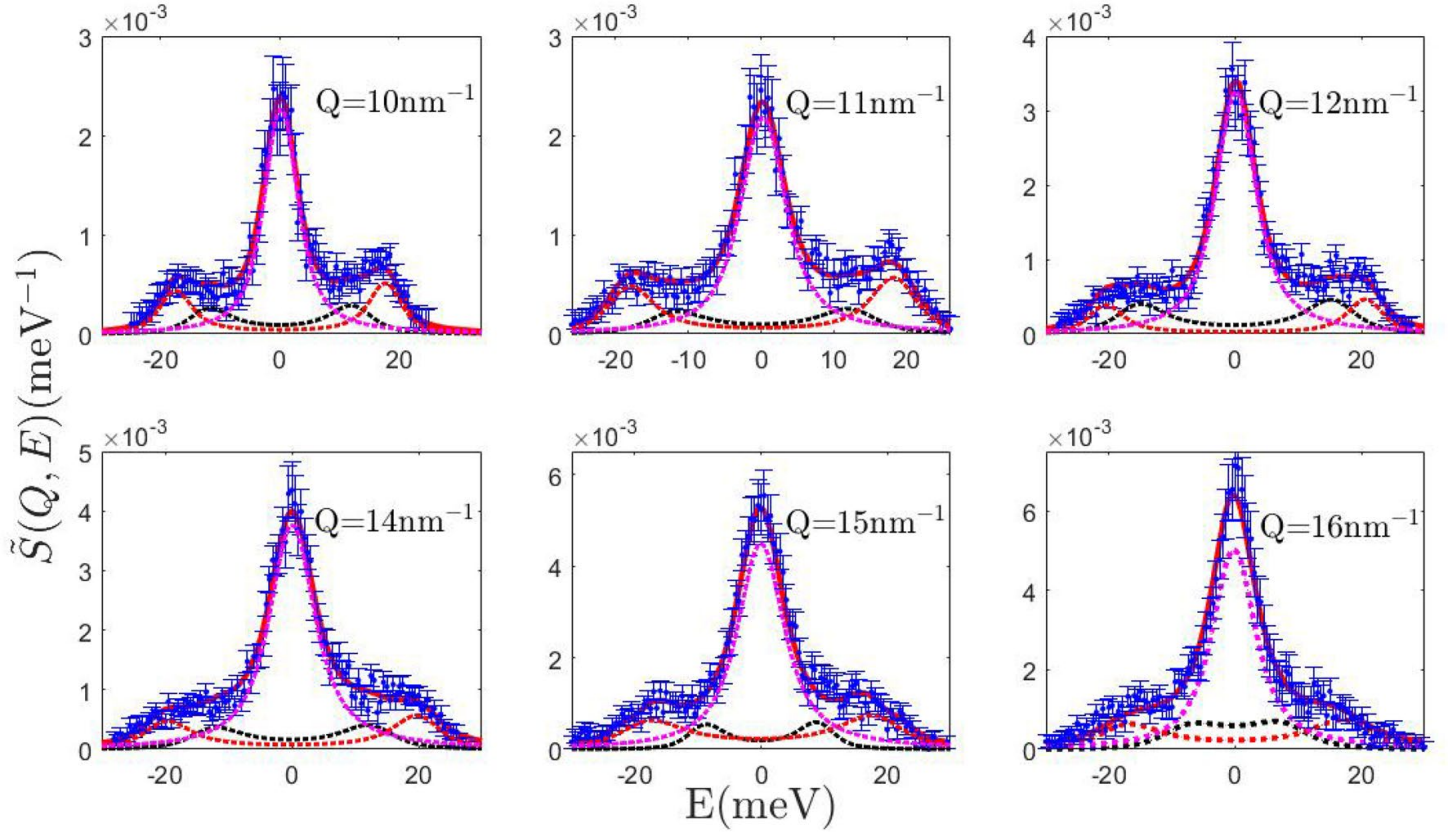
Dynamic structure factor of silver at six selected *Q* values. The experimental data points are affected by resolution broadening. The corresponding best fit (solid red line) obtained by the MCMC-RJ algorithm for the *k* = 2 model has been carried out considering the detailed balance asymmetry and finite resolution. The black and the red dotted lines are the low- and the high-frequency DHO components respectively while the pink dotted line is again the Lorentzian term.

In the attempt of reconciling the outcome of the current analysis with previous literature results on MMs^2–4^, we performed a best-fit of the measured spectral shape with the far less plausible *k* = 2 model option corresponding to a double excitation spectral shape (Fig. 2). This is simply done by withdrawing the RJ option and making the MCMC algorithm run with *k* = 2. Although this model option yields reasonable fits to experimental data (Fig. 5), the Occam razor’s principle causes the Bayesian inference algorithm to privilege model variants containing a minimum number of free parameters over those unnecessarily complex.

**Figure 6:**
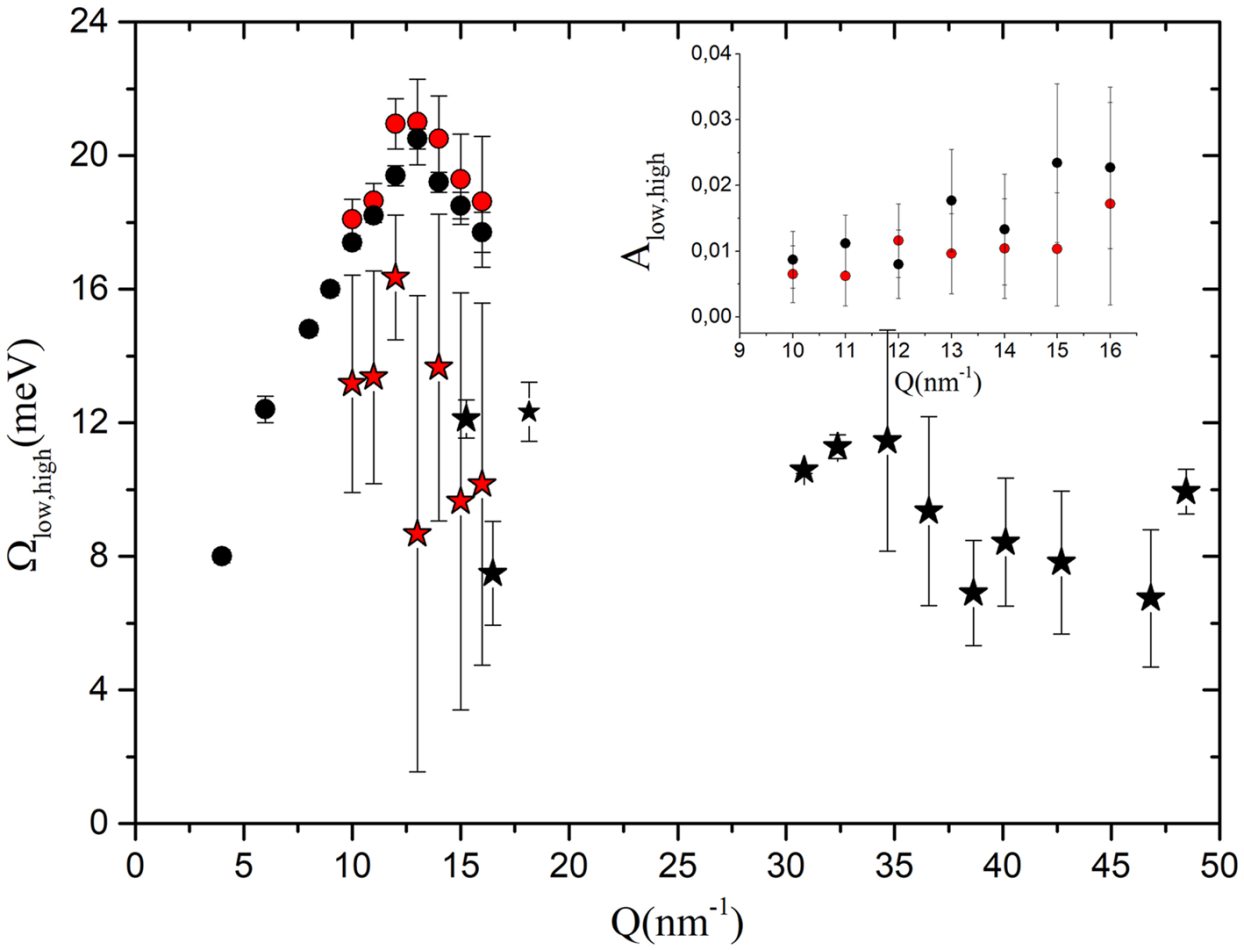
Dispersion curves for the low- and the high-energy modes corresponding to the 2 DHOs model option are reported as red symbols. The frequencies of the longitudinal acoustic mode obtained with the most plausible single DHO model (black circles) is also reported for comparison with the 2-DHO sub-optimal solution (red circles). The black stars represent the frequency of the transverse mode fitted to the AIMD simulations as done in Ref.^23^ and the red stars the frequency of the second mode as from the MCMC algorithm. Inset: areas of the low (red symbols) and high (black symbols) inelastic peaks.

Based on the AIMD simulation outcome, we decided to carry out a double excitation modeling uniquely for the spectra collected in the *Q*, 10–16 $\hbox {nm}^{-1}$ interval. We could be tempted to contemplate as plausible the frequency values for the low-energy mode, particularly for large enough *Q* values. In Fig. 6 we compare the values we found with such modeling with the frequency of the transverse mode derived from the computer simulation in Ref.^23^; for the sake of clarity, we did not include here the longitudinal mode frequencies obtained from the simulation. It clearly appears that uncertainties in the second excitation’s frequencies (red stars) are obviously much larger than those corresponding to a single DHO option, consistently with the low plausibility attributed to the second mode by the Bayesian algorithm.

Finally, data in the inset of Fig. 6 are especially meaningful and worth further comments. The plot compares the relative peak areas of the low and high frequency inelastic excitations, respectively assigned to the transverse and the longitudinal acoustic mode. It clearly emerges that the two parameters attain comparable values in the tested *Q* range, at odds with what suggested by the analysis of AIMD spectra, for which the transverse acoustic mode, if present in that range, is so weak to remain below the detectability threshold. This evident inconsistency with the simulation outcome further undermines the plausibility of a double mode hypothesis for the measured spectral shapes.

**Figure 7:**
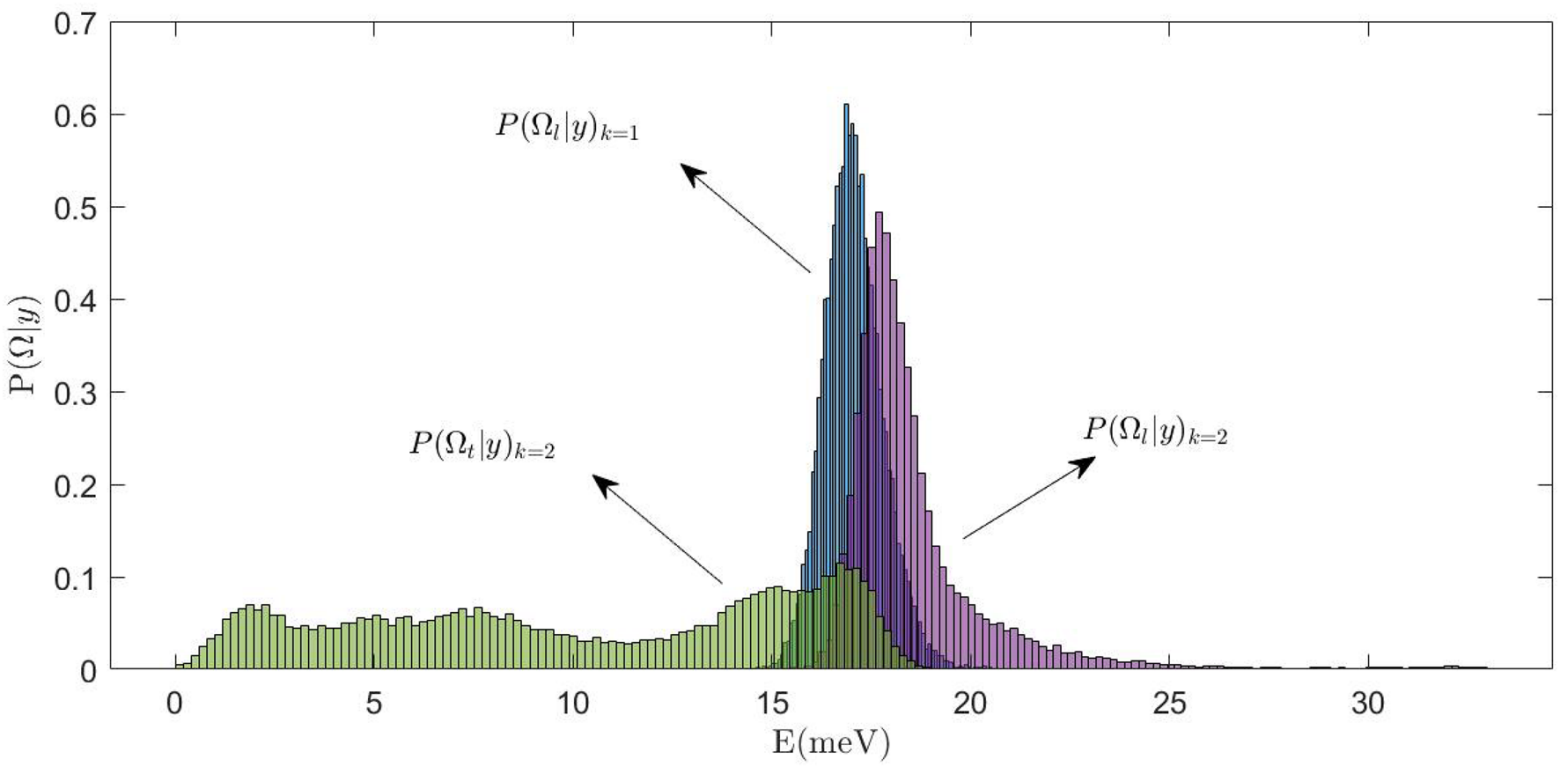
Three posterior distributions from the analysis of the spectrum at *Q* = 16 $\hbox {nm}^{-1}$ are plotted. $P(\Omega _l\vert y)_{k=1}$ (in blue) is the conditional probability for the longitudinal frequency when the model with $k=1$ is chosen. $P(\Omega _{l/t}\vert y)_{k=2}$ are the conditional probabilities for the frequencies which might be associated to the transverse mode (in green) and to the longitudinal mode (in violet) respectively.

To get a more complete picture of the Bayesian inference results, it is worth giving a closer look at Fig. 7, which provides a meaningful example of how the $\Omega _l (Q)$ (*l* for longitudinal) and $\Omega _t (Q)$ (*t* for transverse) posteriors pertaining to either the $k=1$ or the $k=2$ model options compare to each other. Such distributions are derived from the best-fitting of the model to the *Q* = 16 $\hbox {nm}^{-1}$ spectrum.

It appears that whenever the probabilistically more grounded model is chosen, best-fit lineshape parameters are determined with a smaller uncertainty, i.e. they have a posterior sharp, unimodal and symmetrical with respect to the mean. Conversely, when the sub-optimal solution is considered, the high frequency mode still preserves a sharp character (as data evidence for this mode is overwhelming, indeed), yet the position, $\Omega _t (Q)$, of the low-frequency mode, namely the transverse one, is poorly defined, the corresponding density function spreading more or less evenly over a very large energy range, therein somehow resembling a uniform distribution. The best fit value of $\Omega _t (Q)$, namely the one reported in Fig. 5, is just obtained averaging over this energy interval and, obviously, has a large standard deviation. This finding suggests that, despite the overall accuracy of the line shape fitting curve, in the spirit of Bayesian inferential approach, we refrain from claiming any evidence for a well-defined second mode based on the measured spectral profiles.

## Conclusions

We have here discussed the results of a Bayesian inferential analysis of inelastic neutron scattering measurements on liquid silver, aiming to unravel a possible coupling of density fluctuations with shear mode propagation. As opposed to other, more popular, fitting approaches, the one considered here delivers not only best-fitting values of lineshape parameters, but also the related probability distributions conditional to the measurement at hand. Furthermore, it provides a minimally invasive or biased interpretation of the spectral shape, as the number of inelastic modes contributing to it is treated in itself as a free model parameter to be optimized on the basis of available experimental data. Most importantly, this method incorporates the Occam razor principle, which, among competing models satisfactorily accounting for some experimental evidence, privileges the one containing the least number of free parameters, whenever more complex models are not adequately justifiable by the available data. Briefly, we believe that the inferential method described here possesses all prerequisites to provide a reliable answer to the topical question this work aims at addressing: does the terahertz spectrum of a liquid metal bear evidence for a high frequency sample rigidity, as revealed by the onset of a shear propagation mode? The outcome of our analysis on liquid gold and silver indicates that an affirmative answer to this question would fall far beyond the accuracy of the measurements carried out so far on these systems. We cannot exclude that some of past literature works on molten metals might have had slightly different results if Bayesian inference had endowed the lineshape modeling with a probabilistic ground, while protecting it from the risk of an overparametrization. On the other hand, the mentioned computer simulations leave open the possibility that a shear mode could be observed in the spectrum of density fluctuations, if more advanced experimental probe and a better statistical accuracy would enable to spot its presence.

We sincerely hope that elements of wisdom inherent to Bayesian inferential methods will rapidly spread inside our scientific community, hopefully inspiring new powerful and probabilistically grounded lineshape analysis protocols; after all, this is one of the viable scenarios ensuring our field of research a sustainable future.

## Methods

As already mentioned, the Bayesian analysis described in this paper has been carried out on a set of experimental data that had been previously obtained by INS on a liquid silver sample. The experimental setup and, even more important, the various steps of the data analysis that are required to extract the wanted dynamic structure factor from the detected raw count rates, have been described in full detail in Ref.^23^, to which we address the interested reader. We remind here that correction of the raw data includes the subtraction of background and container scattering signals as determined by ancillary measurements. Moreover, both the incoherent and the multiple scattering must be evaluated at best and subtracted, in order to obtain the correct double-differential cross section and, from this, the final *S*(*Q*, *E*). The data points with error bars shown in Fig. 1 are examples of the results of this analysis. A further auxiliary measurement provides the scattering signal of a reference vanadium sample from which the energy resolution is obtained.

We stress that the experiment was carried out using the thermal-neutron Brillouin spectrometer BRISP, which exploits one of the intense neutron beams of the high-flux reactor of the Institute Laue–Langevin (Grenoble, France). In order to cover an appropriate region of the (*Q*, *E*) plane with the small-angle scattering setup required for neutron Brillouin scattering, the incident neutron energy was chosen to be $E_0$ = 83.8 meV, providing an energy resolution of 3.2 meV (FWHM). The Gaussian shape of the BRISP resolution function is essential for a reliable determination of low-frequency features in spectra dominated by an intense central peak, as is typically the case in liquids.

## Supplementary information

Supplementary Informations.
