# Supplementary material for: Searching for a second excitation in the inelastic neutron scattering spectrum of a liquid metal: a Bayesian analysis
Source: Sci Rep. 2021 Jul 7;11:13974. doi: 10.1038/s41598-021-93452-w (PMC8263748; doi:10.1038/s41598-021-93452-w)
Supplement: Supplementary file 1 — Supplementary Informations. [file 41598_2021_93452_MOESM1_ESM.pdf]

Supplementary Information for the Manuscript: Searching for a second excitation in the inelastic neutron scattering spectrum of a liquid metal: a Bayesian analysis

Alessio De Francesco[1,2,+], Ubaldo Bafile[3,+], Alessandro Cunsolo[4,+], Luisa Scaccia[5,+], and Eleonora Guarini[6,+,*]

[1]CNR-IOM & INSIDE@ILL c/o Operative Group in Grenoble (OGG), F-38042 Grenoble, France
[2]Institut Laue-Langevin (ILL), F-38042 Grenoble, France
[3]Consiglio Nazionale delle Ricerche, Istituto di Fisica Applicata ”Nello Carrara”, via Madonna del Piano 10, I-50019 Sesto Fiorentino, Italy
[4]Department of Physics, University of Wisconsin at Madison, 1150 University Avenue, Madison, WI, USA
[5]Dipartimento di Economia e Diritto, Università di Macerata, Via Crescimbeni 20, 62100 Macerata, Italy
[6]Dipartimento di Fisica e Astronomia, Università di Firenze, via G. Sansone 1, I-50019 Sesto Fiorentino, Italy
[*]guarini@fi.infn.it
[+]these authors contributed equally to this work

ABSTRACT

When probed at nanometer and picosecond scales, the properties of a liquid present striking analogies with the one of the corresponding solid, one of the most surprising is the ability of supporting shear wave propagation, as a rigid medium. Although this evidence is being reported by a growing number of terahertz scattering measurements, it remains an open question whether it is universal or rather typical of some liquids only. Furthermore, given its elusive signatures in the scattering signal, the detection of this effect appears as a typical case where an unintentional "bias of confirmation" can mislead experimentalists. We thus decided to use a Bayesian inference approach to achieve a probabilistically grounded and evidence-based lineshape modeling of the Inelastic Neutron Scattering spectra from liquid silver, whose simulated density autocorrelations bear evidence of a shear mode propagation over very short distances. The result of our analysis indicates that the observation of any additional, non-longitudinal, acoustic modes in this simple system goes beyond the accuracy of the used scattering method.

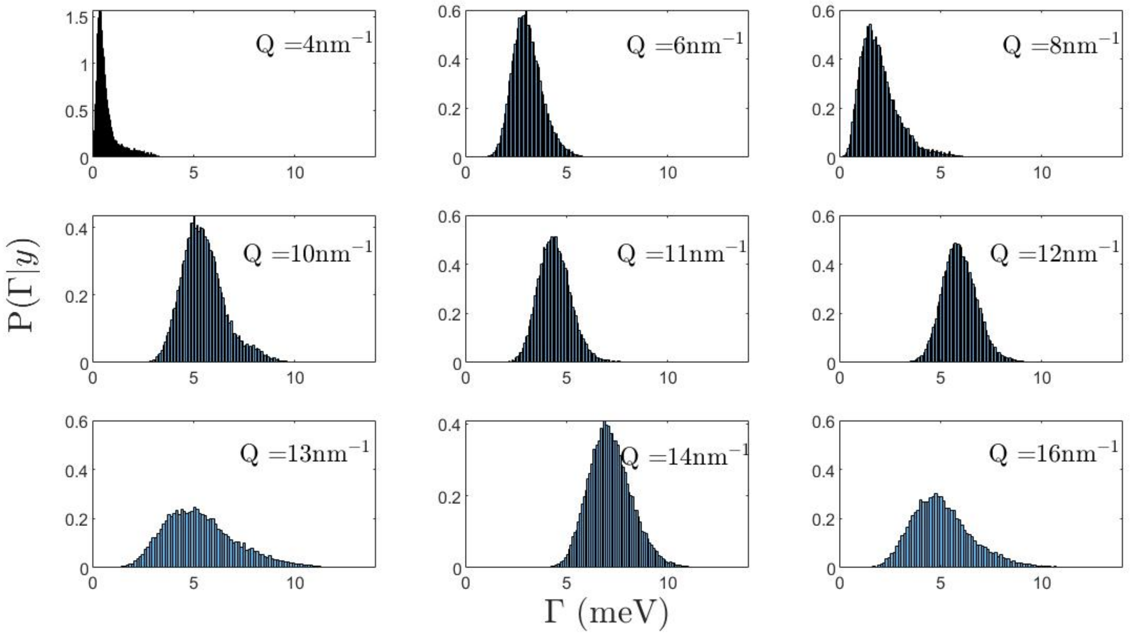

Figure S1. Posterior distribution functions of the damping as derived from the best fit of the spectra at nine selected Q values.

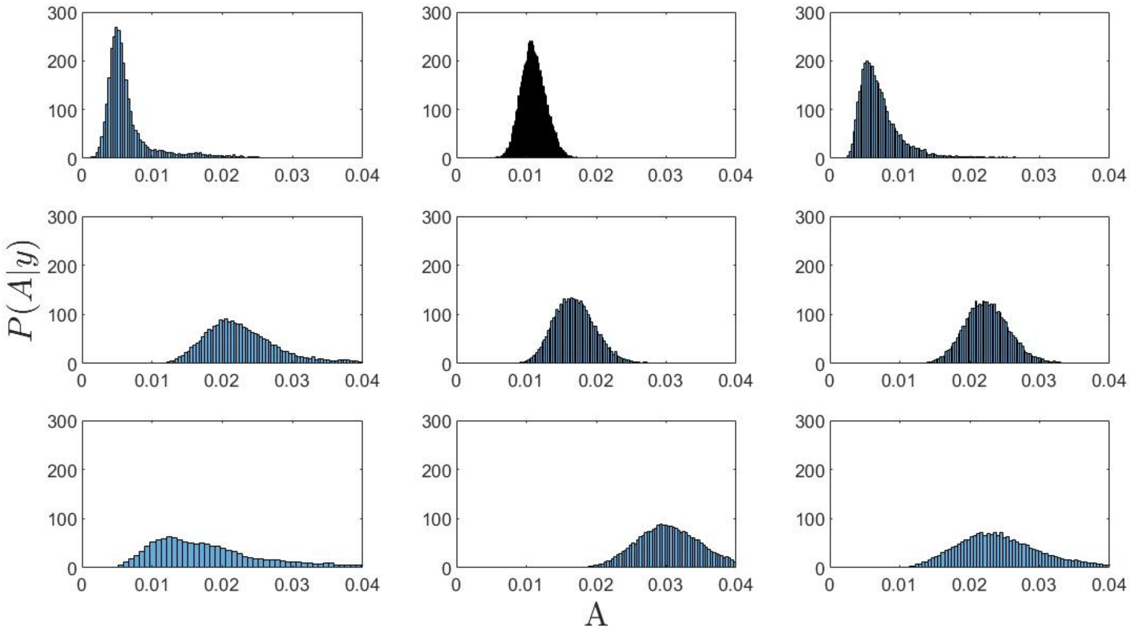

Figure S2. Posterior distribution functions of the peak area of the DHO as derived from the best fit of the spectra at the same selected Q values as in Fig. S1.

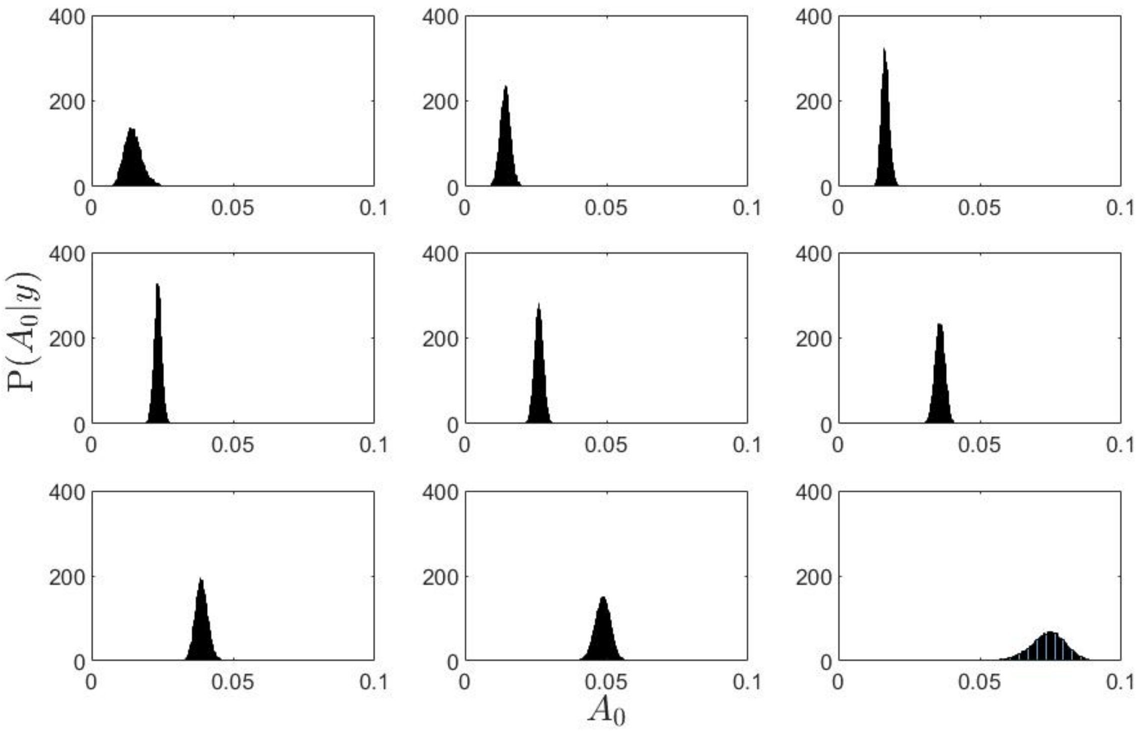

Figure S3. Posterior distribution functions for the amplitude A_0 of the Lorentzian term of the fit function, for the same selected spectra as in Fig. S1.

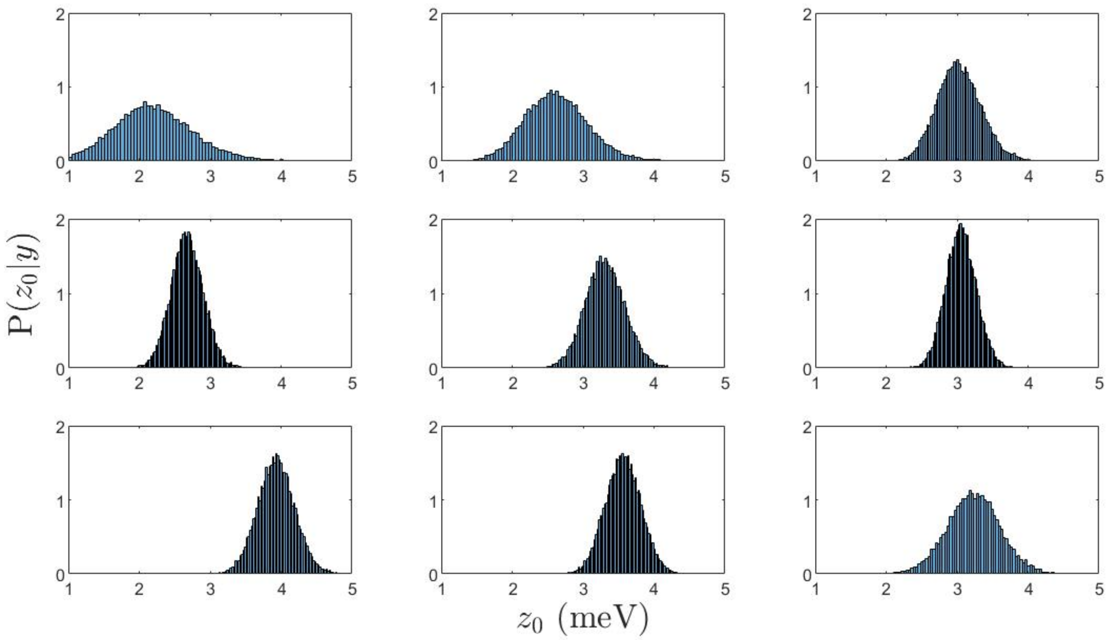

Figure S4. Posterior distribution functions for the half width z_0 of the Lorentzian term of the fit function, for the same selected spectra as in Fig. S1.

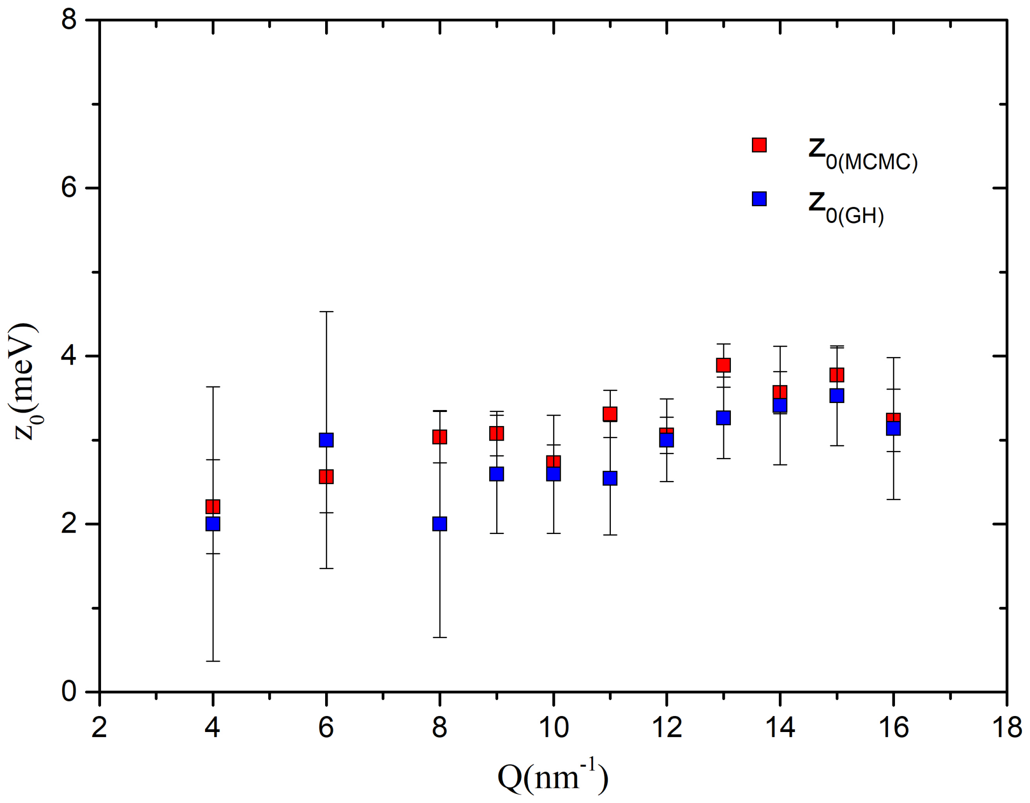

Figure S5. Q dependence of the z_0 parameter as derived from the GH model and MCMC algorithm.
